# Diabetes and the occurrence of infection in primary care: a matched cohort study

**DOI:** 10.1186/s12879-018-2975-2

**Published:** 2018-02-05

**Authors:** Waseem Abu-Ashour, Laurie K Twells, James E Valcour, John-Michael Gamble

**Affiliations:** 1 School of Pharmacy, Health Sciences Centre, Memorial University of Newfoundland, St. John’s, A1B 3V6 Newfoundland and Labrador Canada; 2 Faculty of Medicine, Health Sciences Centre, Memorial University of Newfoundland, St. John’s, A1B 3V6 Newfoundland and Labrador Canada; 3 School of Pharmacy, Faculty of Science, University of Waterloo, Kitchener, N2G 1C5 ON Canada

**Keywords:** Diabetes, Infection, Primary care, Matched cohort, CPCSSN

## Abstract

**Background:**

People with diabetes may be at higher risk for acquiring infections through both glucose-dependent and biologic pathways independent of glycemic control. Our aim was to estimate the association between diabetes and infections occurring in primary care.

**Methods:**

Using the Newfoundland and Labrador Sentinel of the Canadian Primary Care Sentinel Surveillance Network, patients with diabetes ≥18 years between 1 January 2008 and 31 March 2013 were included with at least 1-year of follow-up. We randomly matched each patient with diabetes on the date of study entry with up to 8 controls without diabetes. Primary outcome was the occurrence of ≥1 primary care physician visits for any infectious disease. Secondary outcomes included primary visits for head & neck, respiratory, gastrointestinal, genitourinary, skin and soft tissue, musculoskeletal, and viral infections. Using multivariable conditional logistic regression analysis, we measured the independent association between diabetes and the occurrence of infections.

**Results:**

We identified 1779 patients with diabetes who were matched to 11,066 patients without diabetes. Patients with diabetes were older, had a higher prevalence of comorbidities, and were more often referred to specialists. After adjusting for potential confounders, patients with diabetes had an increased risk of any infection compared to patients without diabetes (adjusted odds ratio = 1.21, 95% confidence interval 1.07–1.37). Skin and soft tissue infections had the strongest association, followed by genitourinary, gastrointestinal, and respiratory infections. Diabetes was not associated with head and neck, musculoskeletal, or viral infections.

**Conclusion:**

Patients with diabetes appear to have an increased risk of certain infections compared to patients without diabetes.

**Electronic supplementary material:**

The online version of this article (10.1186/s12879-018-2975-2) contains supplementary material, which is available to authorized users.

## Background

In 2015 an estimated 415 million people were diagnosed with diabetes mellitus globally. According to the International Diabetes Federation (IDF) this number is expected to rise to more than 640 million people by the year 2040 [1]. Canada is a country that that will be significantly affected by this change. As of 2014, there were an estimated 2 million people aged 12 and older living with diabetes in Canada [2]. Although diabetes is associated with chronic complications of the macrovasculature and microvasculature, other non-traditional complications that include connective tissues disorders and impaired immunity are becoming increasingly recognized [3].

Infection is a relatively frequent reason for hospitalization or a physician office visit in people with diabetes. In fact, about 40% of all people with diabetes have at least one physician claim, and nearly 6% have at least one hospitalization for an infectious disease each year [4]. Moreover, infectious disease contributes to substantial financial costs in people with diabetes. A study conducted in North California estimated the proportion of costs spent on treating complications associated with all types of diabetes across different age groups (< 19 - > 65 years). Costs were categorized by inpatient care, outpatient care (primary care, specialty, emergency, non-physician care), pharmacy and out of plan referrals and claims. They found an excess cost of almost 5 million dollars spent due to infections over one year for people with diabetes compared to people without diabetes [5].

People with diabetes may be more susceptible to infectious disease than those without diabetes. Immunologic research has demonstrated several defects in host immune defense mechanisms in people with diabetes. Phagocytic capabilities of neutrophils are adversely affected by hyperglycemia, including impaired migration, phagocytosis, intracellular killing, and chemotaxis [6, 7]. Besides generalized impairments of immunity, macrovascular disease and microvascular dysfunction may result in compromised local circulation leading to delayed response to infection and impaired wound healing [8]. Unawareness of lower extremity trauma due to sensory neuropathy may result in inadequate attention to minor wounds and subsequent increased infection risk. Incomplete bladder emptying due to autonomic neuropathy permits urinary colonization by microorganisms, where high glucose concentration in the urine promotes the growth of some microorganisms [9].

Despite the aforementioned biologic mechanisms, there are a paucity of studies that have explored the relationship between diabetes and the susceptibility to different types of infections in a primary care setting [4, 10]. Thus, we conducted a matched cohort study in order to measure the relationship between diabetes and the susceptibility to common infections in primary care.

## Methods

### Study design

A matched cohort study using the Newfoundland and Labrador (NL) subset of the Canadian Primary Care Sentinel Surveillance Network (CPCSSN) database was conducted.

### Data source

CPCSSN is Canada’s first multi-disease electronic medical record (EMR)-based surveillance system [11, 12]. In NL, the Atlantic Practice Based Research Network is a group of primary care providers that contribute information to NL sentinel of CPCSSN. For this study, de-identified data was used from the NL-CPCSSN database. Approximately 10% of approximately 500 family physicians within NL contribute data to the CPCSSN database, representing about 45,000 patients. Information available includes patient demographics (e.g., age, sex), health behaviors (smoking), physiological data (e.g., blood pressure) and laboratory data (e.g., HbA1c, fasting glucose, renal function), physician diagnoses, prescription medications, and vaccinations. The sensitivity and specificity of diagnostic algorithms for diabetes in the database is very high with 100% and 99%, respectively [13]. The CPCSSN database has been used for several epidemiologic studies [14, 15].

### Inclusion criteria

The source population for this study included patients with at least one year of history within the NL-CPCSSN database who were 18 years and older between January 1, 2008 and March 31, 2013. Patients with diabetes were identified using the CPCSSN validated algorithm based on International Classification of Disease (ICD-9) billing codes, problem lists, medications, and laboratory values [12] (See Appendix A in Additional file 1). January 1, 2008 or the date a person was diagnosed with diabetes after January 1, 2008 was considered a person’s cohort entry or index date. Controls were selected from patients without diabetes that were alive on the date their matched patient with diabetes entered the cohort. Up to 8 controls were selected for each patient with diabetes and matched on index year. We used a matched cohort design and adjusted for confounders using a standard multivariable regression approach. Given that matching does not necessarily remove bias and no standard practice exists for which variables to match [16, 17], we decided to match on index year only and further adjust for potential confounders using a regression modeling approach. Specifically, we used a multivariable conditional regression model to estimate odds ratios for our primary and secondary outcomes for those with diabetes compared to those without. In addition, we were unable to match on other potential covariates such as age and sex due to limited availability of controls in certain strata. Moreover, we were also interested in explicit testing of effect modification, which we would not be able to do if age and sex were used as matching variables.

### Exclusion criteria

Patients with a diagnosis of malignancy; HIV infection; organ transplantation; use of immunosuppressive medications, ≥ 10 corticosteroids or antibiotics prescriptions in the year prior to the study cohort entry date were excluded from the study [10] (See Appendix B in Additional file 1 for diagnostic codes).

### Outcome measures

The primary outcome was the occurrence of one or more primary care physician visits for any infectious disease during follow-up. The NL-CPCSSN dataset uses ICD-9 codes for all diagnoses (e.g., encounter diagnosis, health condition, and family history). NL-CPCSSN does not have a limit on the number of ICD-9 codes recorded. Infection-related visits to the primary care physician were identified using ICD-9 codes, problem lists, and medications (See Appendix A in Additional file 1).

Secondary outcomes included the number of head and neck infections, respiratory tract infections, gastrointestinal infections, genitourinary tract infections, skin and soft tissue infections, musculoskeletal infections and viral infections (See Appendix A in Additional file 1 for diagnostic and medication codes).

Patients were followed from their index date until March 31, 2014, providing a minimum follow-up period of 1 year. Given multiple episodes of infection occurred throughout follow-up, a new episode was defined if a patient was free of signs or symptoms for a 30-day period. A second episode of the same type of infection occurring > 30 days after the initial episode was considered to be a recurrence (See Appendix C in Additional file 1).

### Statistical analysis

Baseline characteristics among patients with new or existing diabetes were compared to those without diabetes using chi-square tests for categorical variables and t-tests for continuous variables. The same matched cohort was used for all analyses. Crude and adjusted odds ratios were measured using univariate and multivariable conditional logistic regression analyses to determine if diabetes was independently associated with the occurrence of the primary and secondary outcomes. Potential confounding variables included in our statistical model were defined based on biological rationale, clinical experience, those available within the NL-CPCSSN database, and those used in other studies for evaluating diabetes and infection. Specifically, adjustment was done for demographics (age, sex, smoking [missing indicator used for unknown smoking status]), the presence of comorbidities (microvascular disease [nephropathy, neuropathy, retinopathy], macrovascular disease [coronary artery disease, peripheral and cerebral vascular disease], heart failure, respiratory disease, dyslipidemia, fatty liver disease, and obesity), medications (acid inhibitors [e.g., proton pump inhibitors], respiratory system medications [e.g. inhaled corticosteroids], anti-lipids [e.g., statins] and vaccines) and number of infections in the year prior to enrollment in our statistical models (See Appendix C in Additional file 1). Testing for presence of a multiplicative interaction was done between diabetes and age, sex, microvascular and macrovascular disease.

To test the robustness of our primary analysis, sensitivity analyses were conducted. First, restriction of the follow-up period to 1 year was done and the analyses repeated for both the primary and secondary outcomes. Second, the analysis was repeated for the primary outcomes evaluating 1 or more, 2 or more, and 3 or more infections. Third, given the high number of infection recurrences throughout follow-up, a fixed effects conditional Poisson regression model was used to quantify the association between diabetes and the number of infections, whereby adjusted incidence rate ratios were calculated. Fourth, since analysis was conducted adjusting for several covariates, a series of multiple regression models adjusted for a number of selected covariates was done. These key covariates included were age; sex; age and sex; age, sex, microvascular and macrovascular disease, and prior infections. Last, we conducted a stepwise backward selection procedure to select model covariates. Age and sex were forced into the stepwise procedure and other covariates removed if the *p*-value was greater than 0.1. Influential observations were identified by calculating dfbeta’s. Our main results were consistent before and aftern exclusion of highly influential observations (dfbeta > 3/sqrt(n)). All analyses was conducted using Stata 12/MP with a *p*-value of < 0.05 considered statistically significant.

## Results

A total of 1779 patients who had diabetes and 11,066 patients without diabetes were followed for an average of 4.2 years. The average age of the total population was 45 (SD 16) years and the majority were females (59%). Patients with diabetes were on average older, underwent more laboratory tests in the year prior to study entry, were more likely to receive vaccines, acid-suppressing medications and lipid-lowering medications, and were more likely to have existing macrovascular or microvascular disease at study entry compared to controls. Although the number of infections and recurrences before 1-year entry to the study was higher in patients with no diabetes, both groups were similar with regards to the average number of doctor visits (Table 1). Most patients with diabetes were treated with metformin constituting 60.5% of the group, while 22% were on insulin.

**Table 1:** Baseline Characteristics for 12,845 Patients With and Without Diabetes

Variable	Diabetes (*n* = 1779)	Non-Diabetes (*n* = 11,066)	*P* value
Age			< 0.001
Mean (SD)	57.5 (15.6)	43.1 (16.5)	
Median (IQR)	59.0 (20)	41.0 (26)	
Gender n, (%)			
Male	889 (50.0)	4435 (40.1)	< 0.001
Female	890 (50.0)	6631 (59.9)	< 0.001
Doctor Visits (Mean, SD)	3.7 (3.9)	3.3 (3.8)	0.015
Referral To a Specialist, n (%)			
0	1568 (88.1)	9927 (89.7)	0.05
1	133 (7.5)	755 (6.8)	0.338
≥2	78 (4.4)	384 (3.5)	0.064
Infection before 1-year entry, n (%)			
0	1385 (77.8)	6261 (56.6)	< 0.001
1	203 (11.4)	1894 (17.1)	< 0.001
≥2	191 (10.7)	2911 (26.3)	< 0.001
Lab Tests (Mean, SD)	14.5 (46.3)	10.6 (33.2)	< 0.001
Vaccines, n (%)	251 (14.1)	1202 (10.9)	< 0.001
Acid-suppressing Medications, n (%)	306 (17.2)	983 (8.9)	< 0.001
Respiratory Medications, n (%)	173 (9.72)	1140 (10.3)	0.482
Lipid-lowering Medications, n (%)	716 (40.2)	653 (5.9)	< 0.001
Microvascular Disease, n (%)	29 (1.6)	23 (0.2)	< 0.001
Macrovascular Disease, n (%)	76 (4.3)	111 (1)	< 0.001
Heart failure, n (%)	19 (1.1)	16 (0.1)	< 0.001
Fatty Liver, n (%)	10 (0.6)	12 (0.1)	< 0.001
Obesity, n (%)	61 (3.4)	129 (1.2)	< 0.001
Respiratory Disease, n (%)	236 (13.3)	2273 (20.5)	< 0.001
Follow up time, years (Mean, SD)	4.2 (1.7)	4.2 (1.7)	0.788

The proportion of patients with diabetes who had one or more primary care visits for an infection was similar to patients without diabetes (57% vs. 58%). Proportions were also comparable between groups within a 1-year follow-up period (33% vs. 32%) (Table 2). The rate of infections per 100 person-years (PYs) was similar between patients with diabetes and patients without (42.7 per 100PYs vs. 42.5 per 100PYs) (Table 3). Tables 2 and 3 display the proportions and rates for specific types of infections. Respiratory tract infections were the most common infections reported.

**Table 2:** Number of Patients With and Without Diabetes Over the Full and 1-Year Follow-up Periods Experiencing One or More Infections

Infection Type	Full Follow-up Period			1-year Follow-up period		
	Diabetes (n = 1779) n (%)	Non-Diabetes (n = 11,066) n (%)	*P* value	Diabetes (n = 1779) n (%)	Non-Diabetes (n = 11,066) n (%)	*P* value
Any Infection	1012 (56.9)	6428 (58.1)	0.354	584 (32.8)	3500 (31.6)	0.327
Head & Neck	118 (6.6)	817 (7.4)	0.280	39 (2.2)	298 (2.7)	0.252
Respiratory	462 (26.0)	3266 (29.5)	0.002	210 (11.8)	1381 (12.5)	0.445
Gastrointestinal	151 (8.5)	694 (6.3)	< 0.001	69 (3.9)	245 (2.2)	< 0.001
Genitourinary	203 (11.4)	1172 (10.6)	0.319	93 (5.2)	438 (4.0)	0.015
Skin & Soft Tissue	199 (11.2)	896 (8.1)	< 0.001	78 (4.4)	269 (2.4)	< 0.001
Musculoskeletal	185 (10.4)	1189 (10.7)	0.692	62 (3.5)	388 (3.5)	1.000
Viral	84 (4.7)	428 (3.9)	0.100	30 (1.7)	130 (1.2)	0.091

**Table 3:** Infections Per 100 Person-Years in Patients With and Without Diabetes Over the Full and 1-year Follow-up Periods

Infection Type	Full Study Period (Mean, SD)			1-year study period (Mean, SD)		
	Diabetes	Non-Diabetes	*P* value	Diabetes	Non-Diabetes	*P* value
Any Infection	42.7 (63.0)	42.5 (59.9)	0.895	54.7 (99.3)	50.2 (94.6)	0.077
Head & Neck	2.1 (11.5)	2.2 (9.7)	0.771	2.6 (18.2)	3.0 (19.5)	0.318
Respiratory	13.4 (36.3)	13.9 (30.0)	0.589	16.2 (52.2)	16.3 (49.7)	0.943
Gastrointestinal	3.4 (15.0)	2.2 (11.1)	0.001	4.9 (27.9)	2.7 (19.4)	0.001
Genitourinary	4.8 (18.0)	4.2 (16.5)	0.148	6.9 (33.1)	4.9 (26.7)	0.016
Skin & Soft Tissue	4.1 (15.3)	2.4 (10.5)	< 0.001	5.2 (26.3)	2.6 (17.4)	< 0.001
Musculoskeletal	3.7 (14.4)	3.8 (14.5)	0.789	4.3 (25.1)	4.0 (22.3)	0.617
Viral	1.4 (7.1)	1.1 (6.6)	0.083	1.7 (13.5)	1.2 (11.4)	0.122

**Table 4:** Odds Ratio (OR) Between Diabetes and Infection Over the Full Follow-up Period: Crude and Adjusted Results

Type of Infection	Crude OR	95%CI	*P* value	Adjusted OR	95%CI	*P* value
Any Infection	0.96	0.86–1.06	0.399	1.21	1.07–1.37	0.002
Head & Neck	0.90	0.74–1.11	0.328	1.13	0.89–1.43	0.313
Respiratory	0.84	0.75–0.94	0.003	1.30	1.13–1.48	< 0.001
Gastrointestinal	1.38	1.15–1.67	< 0.001	1.40	1.12–1.75	0.003
Genitourinary	1.10	0.94–1.29	0.238	1.48	1.22–1.81	< 0.001
Skin & Soft Tissue	1.43	1.21–1.69	< 0.001	1.66	1.37–2.02	< 0.001
Musculoskeletal	0.97	0.82–1.14	0.714	1.05	0.87–1.28	0.598
Viral	1.21	0.95–1.55	0.115	1.15	0.86–1.54	0.351

Adjusted for age, sex, smoking, comorbidities (microvascular disease [nephropathy, neuropathy, retinopathy], macrovascular disease [coronary artery disease, peripheral and cerebral vascular disease], heart failure, respiratory disease, dyslipidemia, fatty liver disease, and obesity), medications (acid inhibitors, respiratory system medications, anti-lipids and vaccines) and number of infections in the year prior to enrollment

After controlling for potential confounding, patients with diabetes had an increased odds of any infection compared to patients without diabetes (adjusted odds ratio (aOR) = 1.21, 95%CI 1.07–1.37, *P* value = 0.002) (Table 4). There was no significant association between diabetes and head and neck infections (aOR = 1.13, 95%CI 0.89–1.43, *P* value = 0.313). However, the risk in patients with diabetes was increased for respiratory infections (aOR = 1.30, 95%CI 1.13–1.48, *P* value< 0.001), gastrointestinal infections (aOR =1.40, 95%CI 1.12–1.75, *P* value = 0.003), genitourinary infections (aOR = 1.48, 95%CI 1.22–1.81, *P* value< 0.001), and skin and soft tissue infections (aOR = 1.66, 95%CI 1.37–2.02, *P* value< 0.001). There were no significant differences when examining musculoskeletal (aOR = 1.05, 95%CI 0.87–1.28, *P* value = 0.598) or viral infections (aOR = 1.15, 95%CI 0.86–1.54, *P* value = 0.351).

There was a significant interaction between diabetes and age for respiratory infections (likelihood ratio test for interaction *p* < 0.001) and viral infections (likelihood ratio test for interaction *p* = 0.006). A stronger association was found between diabetes and respiratory infections (aOR = 1.24, 95%CI 1.08–1.42, *P* value = 0.002) and viral infections (aOR = 1.48, 95%CI 1.07–2.06, P value = 0.018) in patients ≥65; however, in patients < 65 years there was no association observed (Respiratory infection: aOR = 1.07, 95%CI 0.91–1.25, *P* value = 0.397; Viral infection: aOR = 1.15, 95%CI 0.86–1.53, P value = 0.336).

Our findings were consistent when examining the number of infections over a 1-year period (See Appendix D in Additional file 1). Recurrence of any infection was also calculated in the study population for ≥1, ≥2 and ≥3 infections (Table 5). There was a consistent increase in the odds with number of recurrences. The adjusted odds ratio for ≥2 recurrent infections was 1.32 (95%CI 1.14–1.52, *P* value = < 0.001), and 1.53 (95%CI 1.29–1.83, *P* value = < 0.001) for ≥3 recurrent infections. In addition, through a fixed effects conditional Poisson regression model, an increased incidence rate ratio (IRR) of any infection was found in patients with diabetes compared to those without diabetes (adjusted IRR = 1.25, 95%CI 1.20–1.31, *P* value< 0.001). When we conducted multiple regression models adjusted for selected covariates and a backward stepwise regression procedure, we found that having prior visits for an infectious disease was a highly influential covariate (See Appendix E in Additional file 1).

**Table 5:** Odds Ratio (OR) Between Diabetes and Any Infection Recurrence: Crude and Adjusted Results

Recurrences	Crude OR	95%CI	P value	Adjusted OR	95%CI	*P* value
≥1 Infection	0.96	0.86–1.06	0.399	1.21	1.07–1.37	0.002
≥2 Infection	0.92	0.82–1.04	0.196	1.32	1.14–1.52	< 0.001
≥3 Infection	0.99	0.86–1.13	0.872	1.53	1.29–1.83	< 0.001

Adjusted for age, sex, smoking, comorbidities (microvascular disease [nephropathy, neuropathy, retinopathy], macrovascular disease [coronary artery disease, peripheral and cerebral vascular disease], heart failure, respiratory disease, dyslipidemia, fatty liver disease, and obesity), medications (acid inhibitors, respiratory system medications, anti-lipids and vaccines) and number of infections in the year prior to enrollment

## Discussion

In the current study, patients with diabetes had a 21% increased chance of developing a new infection compared to patients without diabetes in primary care practices over an average of approximately 4 years. The positive association between diabetes and the development of a community-acquired infection was observed within the first year of follow-up (36% relative odds increase). The strongest magnitude of association was found for skin and soft tissue infections (66% relative odds), followed by genitourinary (48% relative odds increase), gastrointestinal (40% relative odds increase), and respiratory infections (30% relative odds). A consistent, slightly stronger, measure of association was observed in the one year follow up. However, no association was present for head and neck, musculoskeletal and viral infections. A graded response was also observed for an increasing number of infections. Interestingly, we found that the number of infections and recurrences before 1-year entry to the study was higher in patients with no diabetes. Higher crude rates of infection in patients without diabetes may be due to the fact that they were on average younger than patients with diabetes [18].

Few studies have explored the relationship between diabetes and the occurrence of general infections in a primary care setting [4, 19–23]. When comparing our results with other studies that examined this relationship in a primary care setting, they were consistent with most authors who have found increased rates of infections in patients with diabetes compared to patients without [4]. For example, Shah and Hux examined the association between diabetes and different types of infections using administrative databases from Ontario, Canada [4]. They quantified the risk of infections in patients with diabetes using an administrative dataset over a relatively short study duration (1 year), and also found an increased risk of infectious disease in patients with diabetes, with an even higher risk ratio of infectious disease related hospitalization. Another cohort study conducted in Japan examined risk factors for infection and concluded that diabetes was one of the important risk factors. Using hospital records and community data the authors reported more than double the risk of infection in patients with diabetes compared to those without [20]. The findings of this study may not be generalizable to primary care patients as the study population had autoimmune diseases. In addition, important confounders that may have affected the outcome, such as corticosteroid therapy, were not taken into consideration. A case-control study that was consistent with our findings found more than double the risk of infection in patients with diabetes compared to patients without diabetes [21]. Shortcomings of this study that should be noted are the small study population and a reliance on self-reported data. In contrast, Lipsky et al. studied risk factors for acquiring pneumococcal infections in a general medical clinic and found that diabetes was not a risk factor for developing pneumococcal infections [23].

Results of our secondary outcomes were consistent with other studies that looked at skin and soft tissue infections [4, 10, 24–27] with a magnitude of association ranging from [aOR = 1.3–3.7], respiratory infections [4, 10, 28–31] [aOR = 1.2–4.7], genitourinary infections [aOR = 1.2–2.8] [4, 10, 32–35], and gastrointestinal infections [4, 24, 36] [aOR = 1.3–1.5]. Despite shortcomings of these studies including specific populations [25, 26, 28, 32], self-reported symptoms [24], potential misclassification [28, 29, 31, 33] or selection bias [27] and the reliance on hospital records [4, 27, 30], their results were consistent with the findings from this study.

There was no difference in the risk of head and neck, musculoskeletal, and viral infections in patients with diabetes and patients without, although a few studies have found an increased risk of these types of infections [4, 10, 37–40].

### Limitations

This study has several limitations. First, patients with diabetes and control patients were different at baseline with regards to some risk factors (e.g., presence of comorbiditiess) that may have confounded the observed association between diabetes and infection. However, to minimize confounding, adjustment was carried out for known differences between the comparison groups using multivariable analysis. Furthermore, if certain macrovascular or microvascular pathologies are involved in the causal pathway between diabetes and developing certain infections, it is possible that by adjusting for macrovascular and microvascular complications in our analysis that we removed a portion of this effect. More advanced models that handle both time dependent confounding and mediations (e.g. marginal structural models) could be used to try and tease out these effects; however, more knowledge of the mediating pathway and a larger dataset would be required to explore the mediating effects of macro and microvascular complications. Second, due to the fact that electronic medical records from routine clinical care were used to define the diagnosis of diabetes, the potential for misclassification of the diabetes (i.e., exposure) exists. Patients with a true diagnosis of diabetes may have been misclassified as not having the disease. Moreover, since over one-third of diabetes is undiagnosed in the community [41], the true number of patients with diabetes may have been underestimated. Alternatively, misclassification for our outcome, infection, may have also occurred. The ability to capture all patients with an infection and assessing some types of infection occurrence in the general population setting is difficult. Some infections, such as viral, genital or other infections, are often treated by self-care, i.e., using products available over-the counter, symptomatic treatment, or no treatment at all. In addition, as medical records from general practice were used to identify the occurrence of infections; it is possible that infections encountered in a hospital setting or treated at a specialist clinic may not be captured. Thus, ascertaining such cases via clinical records may underestimate incidence. In addition, information was not available for vital status or hospitalizations, which could have led to not capturing patients who had an infection but were hospitalized without the knowledge of their primary care physician. Moreover, vitals can provide a clearer picture of occurrence, worsening or improvement of an infection. Third, increased physician contact for patients with diabetes or differences in misclassification may have resulted in diagnostic bias. Physicians may have focused on and followed more closely, or treated patients with diabetes differently due to regular clinical encounters. Fourth, comparison between different types of diabetes and the association of infection was not investigated here. Type of diabetes is a possible effect modifier that could have affected the magnitude of measure of association; however, dysglycemia is a common consequence across all types of diabetes. Moreover, several studies that examined types of diabetes and infection found consistent results with regards to the presence of association [10, 33, 42]. In addition, prevalent and incident cases of diabetes were included in our cohort. The difference in their glycemic control could affect the infection risk in these individuals. Fifth, although age was adjusted for in the model, residual confounding may still be present. Matching by age was impractical due to insufficient numbers. Finally using the CPCSSN dataset, although designed for epidemiological research purposes, may have limited our generalizability. This dataset only includes physicians using an electronic medical record, who may differ from other physicians who do not use electronic medical records.

## Conclusion

Our study supports a significant association between diabetes and specific community-acquired infections including skin and soft tissues, gastrointestinal, genitourinary and respiratory infections. The implications of glucose control via antidiabetic medications on the risk of these infections remains to be determined.

## Additional file

Additional file 1: Appendices A through E. (DOCX 22 kb)

## Abbreviations

aOR: adjusted odds ratio
CI: confidence interval.
CPCSSN: Canadian Primary Care Sentinel Surveillance Network
EMR: electronic medical record
HIV: human immunodeficiency virus
ICD: international classification of diseases
IDF: international diabetes federation
NL: Newfoundland and Labrador
PYs: person-years
SD: standard deviation
