# Supplementary material for: Diabetes and the occurrence of infection in primary care: a matched cohort study
Source: BMC Infect Dis. 2018 Feb 5;18:67. doi: 10.1186/s12879-018-2975-2 (PMC5800043; doi:10.1186/s12879-018-2975-2)
Supplement: Additional file 1: — Appendices A through E. (DOCX 22 kb) [file 12879_2018_2975_MOESM1_ESM.docx]

**Appendices:**

**Appendix A: Exposure and Outcome Case Definitions**

| **Exposure/Outcome** | | | | | **Classification of Variables** |
| --- | --- | --- | --- | --- | --- |
| Diabetes | | | | | CPCSSN Validated Case Definition using problem list (Diabetes, NIDDM, DM), Diagnostic codes (ICD-9: 250.0-250.9),  medication list (insulin, oral antidiabetic agents, ATC code A10), and  lab test results (HbA1c>7%, fasting BS>7) |
| Infection | **Any infection-Related Visit** | |  |  | Any record in EMR for any infection plus ICD-9 codes: 001-139, 320-326, 391.1, 391.2, 421, 451, 685, 461.0-461.3, 461.8, 461.9, 473.0-473.3, 473.8, 473.9, 460, 472.2, 462, 472.1, 463, 474.00, 464.3, 464.5, 464.0, 476.0, 464.2, 476.1, 464.1, 491.8, 466, 490, 491, 480-486, 510 + (041.0-041.9), 010-018, 590.0-590.3, 590.8, 590.9, 595, 597, 601.0, 601.1, 601.9, 604, 607.1, 112.2, 611.0, 614.0-614.5, 614.7, 615.9, 616.0, 616.1, 616.81, 090-099, V02.7, 647.1, 110-118, 680, 681-682, 683, 684, 686, 728, 727.00, 727.02, 727.05. 727.06, 729.4, 730.0-730.2, 730.3, 382.2, 711.0-711.9, 003, 005, 004, 006, 009.0, 009.1, 531-535, 540-543, 567, 370.3, 370.4, 372.0-372.3, 373.0, 373.1, 373.4-373.6, 373.8, 373.9, 076, 380.0, 380.1, 381.0-381.5, 382.0-382.9, 384.0, 384.1, 383.0, 383.1, 383.9, 052, 053, 054, 055, 056, 072, 487, 488, 070, J01, J02, J04, J05, D01, D06, G01, P01, P02, P03, A07A |
| Infection | **Respiratory Tract Infections** | | **Upper respiratory tract infection** | Sinusitis | Any record in EMR plus ICD-9: 461.0-461.3, 461.8, 461.9, 473.0-473.3, 473.8, 473.9 |
|  |  |  |  | Naso-pharyngitis | Any record in EMR plus ICD-9: 460, 472.2 |
|  |  |  |  | Pharyngitis | Any record in EMR plus ICD-9: 462, 472.1 |
|  |  |  |  | Tonsillitis | Any record in EMR plus ICD-9: 463, 474.00 |
|  |  |  |  | Epiglottitis | Any record in EMR plus ICD-9: 464.3, 464.5 |
|  |  |  |  | Laryngitis | Any record in EMR plus ICD-9: 464.0, 476.0 |
|  |  |  |  | Laryngo-tracheitis | Any record in EMR plus ICD-9: 464.2, 476.1 |
|  |  |  |  | Tracheitis | Any record in EMR plus ICD-9: 464.1, 491.8 |
|  |  |  | **Lower respiratory tract infection** | Bronchitis | Any record in EMR plus ICD-9: 466, 490, 491 |
|  |  |  |  | Pneumonia | Any record in EMR plus ICD-9: 480-486 |
|  |  |  |  | Empyema | Any record in EMR plus ICD-9: 510 + (041.0-041.9) |
|  |  |  |  | Tuberculosis | Any record in EMR plus ICD-9: 010-018 |
|  |  | |  |  |  |
|  | **Genitourinary Tract Infection** | | **Urinary tract infection** | Pyelonephritis | Any record in EMR plus ICD-9: 590.0-590.3, 590.8, 590.9 |
|  |  |  |  | Cystitis | Any record in EMR plus ICD-9: 595 |
|  |  |  |  | Urethritis | Any record in EMR plus ICD-9: 597 |
|  |  |  |  | Prostatitis | Any record in EMR plus ICD-9: 601.0, 601.1, 601.9 |
|  |  |  | **Genital infection** | Epididymitis | Any record in EMR plus ICD-9: 604 |
|  |  |  |  | Balanitis | Any record in EMR plus ICD-9: 607.1, 112.2 |
|  |  |  |  | Pelvic Inflammatory Disease | Any record in EMR plus ICD-9: 611.0, 614.0-614.5, 614.7, 615.9, 616.0, 616.1, 616.81 |
|  |  |  |  | Syphilis | Any record in EMR plus ICD-9: 090-099 |
|  |  |  |  | Gonorrhea | Any record in EMR plus ICD-9: V02.7, 647.1 |
|  |  | |  |  |  |
|  | **Skin and Soft Tissue Infection** | | | Mycoses | Any record in EMR plus ICD-9: 110-118 |
|  |  |  |  | Carbuncle and Furuncle | Any record in EMR plus ICD-9: 680 |
|  |  |  |  | Cellulitis | Any record in EMR plus ICD-9: 681-682 |
|  |  |  |  | Lymphadenitis | Any record in EMR plus ICD-9: 683 |
|  |  |  |  | Impetigo | Any record in EMR plus ICD-9: 684 |
|  |  |  |  | Other | Any record in EMR plus ICD-9: 686 |
|  |  |  | |  |  |
|  | **Musculoskeletal Infection** | | | Myositis | Any record in EMR plus ICD-9: 728 |
|  |  |  |  | Synovitis | Any record in EMR plus ICD-9: 727.00, 727.02, 727.05. 727.06 |
|  |  |  |  | Fasciitis | Any record in EMR plus ICD-9: 729.4 |
|  |  |  |  | Osteomyelitis | Any record in EMR plus ICD-9: 730.0-730.2 |
|  |  |  |  | Periostitis | Any record in EMR plus ICD-9: 730.3 |
|  |  |  |  | Petrositis | Any record in EMR plus ICD-9: 382.2 |
|  |  |  |  | Arthropathy Associated Infection | Any record in EMR plus ICD-9: 711.0-711.9 |
|  |  |  | |  |  |
|  | **Gastrointestinal Infection** | | | Salmonela (food poisoning) | Any record in EMR plus ICD-9: 003, 005 |
|  |  |  |  | Shigellosis | Any record in EMR plus ICD-9: 004 |
|  |  |  |  | Amebiasis | Any record in EMR plus ICD-9: 006 |
|  |  |  |  | Gastroenteritis | Any record in EMR plus ICD-9: 009.0, 009.1 |
|  |  |  |  | Ulcer | Any record in EMR plus ICD-9: 531-535 |
|  |  |  |  | Appendicitis | Any record in EMR plus ICD-9: 540-543 |
|  |  |  |  | Peritonitis | Any record in EMR plus ICD-9: 567 |
|  |  |  | |  |  |
|  | **Head & Neck Infections** | | | Conjunctivitis | Any record in EMR plus ICD-9: 370.3, 370.4, 372.0-372.3 |
|  |  |  |  | Blepharitis | Any record in EMR plus ICD-9: 373.0, 373.1, 373.4-373.6, 373.8, 373.9 |
|  |  |  |  | Trachoma | Any record in EMR plus ICD-9: 076 |
|  |  |  |  | Otitis Externa | Any record in EMR plus ICD-9: 380.0, 380.1 |
|  |  |  |  | Otitis Media | Any record in EMR plus ICD-9: 381.0-381.5, 382.0-382.9 |
|  |  |  |  | Myringitis | Any record in EMR plus ICD-9: 384.0, 384.1 |
|  |  |  |  | Mastoditis | Any record in EMR plus ICD-9: 383.0, 383.1, 383.9 |
|  |  |  | |  |  |
|  | **Viral Infections** | | | Chickenpox | Any record in EMR plus ICD-9: 052 |
|  |  |  |  | Herpes Zoster | Any record in EMR plus ICD-9: 053 |
|  |  |  |  | Herpes Simplex | Any record in EMR plus ICD-9: 054 |
|  |  |  |  | Measles | Any record in EMR plus ICD-9: 055 |
|  |  |  |  | Rubella | Any record in EMR plus ICD-9: 056 |
|  |  |  |  | Mumps | Any record in EMR plus ICD-9: 072 |
|  |  |  |  | Influenza | Any record in EMR plus ICD-9: 487, 488 |
|  |  |  |  | Hepatitis | Any record in EMR plus ICD-9: 070 |
|  |  |  | |  |  |
|  | **Medications** | | | Anti-infectives | Any record in EMR plus ATC code: J01, J02, J04, J05, D01, D06, G01, P01, P02, P03, A07A |

**Appendix B: Diagnostic Codes for Exclusion**

| **Category** | | **Classification of Variables** |
| --- | --- | --- |
| Neoplasms | | Any record in EMR plus ICD-9: 140-239 |
| AIDS/HIV | | Any record in EMR plus ICD-9: 042-044 |
| Organ Transplant | Kidney | Any record in EMR plus ICD-9: 55.6 |
|  | Liver | Any record in EMR plus ICD-9: 50.51 |
|  | Heart | Any record in EMR plus ICD-9: 37.52, 37.60, 37.65-37.67 |
| Medication use | Immunosuppressant | Any record in EMR plus ATC code: L04 |
|  | Corticosteroids | Any record in EMR plus ATC code: H02 |

**Appendix C: Case Definitions for Potential Confounders and Effect Modifiers**

| **Category** | **Variable Name** | | **Classification of Variable** |
| --- | --- | --- | --- |
| Demographic | ID | | De-identified ID |
|  | Age | | Birth year |
|  | Sex | | Sex |
|  | Smoking | | Smoking |
| Lab Values | HbA1c | | HbA1c |
|  | Temperature >37.5 C | | Temperature |
|  | WBC > 10,000 mcL | | WBC count |
|  | Fasting Glucose | | Fasting Glucose |
|  | BMI | | BMI |
|  | Lipids | | Lipids |
|  | Urine albumin creatinine ratio | | Urine albumin creatinine ratio |
| Health Services Utilization | Number of primary care physician visits | | Number of visits |
|  | Referrals to specialist | | Number of referrals |
|  | Number of lab tests ordered | | Number of lab tests |
| Medications | Anti-diabetic Medications | | Any record in EMR plus ATC code: A10A, A10B |
|  | Immunosuppressant | | Any record in EMR plus ATC code: L04 |
|  | Corticosteroids | | Any record in EMR plus ATC code: H02 |
|  | Immunoglobulins | | Any record in EMR plus ATC code: J06 |
|  | Vaccines | | Any record in EMR plus ATC code: J07 |
|  | Drugs for Acid-related Disorders | | Any record in EMR plus ATC code: A02A, A02B, A02X |
|  | Respiratory System Agents | | Any record in EMR plus ATC code: R01-R07 |
|  | Lipid Modifying Agents | | Any record in EMR plus ATC code: C10 |
| Diabetes Comorbidities | Microvascular | Nephropathy | Any record in EMR plus ICD-9: 250.40-250.43, 583.81, 581.81 |
|  |  | Neuropathy | Any record in EMR plus ICD-9: 250.60-250.63, 337.1, 357.2, 536.3, 353.5 |
|  |  | Retinopathy | Any record in EMR plus ICD-9: 250.50-250.53, 362.00-362.07, 369.00-369.90, 366.41, 265.41 |
|  | Macrovascular | Peripheral vascular disease | Any record in EMR plus ICD-9: 443.9, 441, 785.4, V43.4, 38.48 |
|  |  | Cerebrovascular disease | Any record in EMR plus ICD-9: 430-438 |
|  |  | Myocardial infarction | Any record in EMR plus ICD-9: 410, 412 |
|  |  | Congestive heart failure | Any record in EMR plus ICD-9: 428 |
|  | Non-Alcoholic Fatty Liver Disease | | Any record in EMR plus ICD-9: 571.8 |
|  | Obesity | | Any record in EMR plus ICD-9: 278, 783.1, V77.8 |
| Respiratory disease |  | | Any record in EMR plus ICD-9: 460-519 |

**Appendix D:** **Odds Ratio (OR) Between Diabetes and Infection Over 1-Year Period: Crude and Adjusted Results**

| **Type of Infection** | **Crude OR** | **95%CI** | **P value** | **Adjusted OR** | **95%CI** | **P value** |
| --- | --- | --- | --- | --- | --- | --- |
| Any Infection | 1.06 | 0.95-1.18 | 0.290 | 1.36 | 1.20-1.54 | <0.001 |
| Head & Neck | 0.83 | 0.59-1.17 | 0.285 | 1.14 | 0.77-1.71 | 0.509 |
| Respiratory | 0.94 | 0.81-1.10 | 0.470 | 1.40 | 1.17-1.69 | <0.001 |
| Gastrointestinal | 1.78 | 1.36-2.34 | <0.001 | 1.70 | 1.21-2.39 | 0.002 |
| Genitourinary | 1.34 | 1.06-1.69 | 0.013 | 1.91 | 1.42-2.56 | <0.001 |
| Skin & Soft Tissue | 1.81 | 1.40-2.34 | <0.001 | 1.99 | 1.45-2.74 | <0.001 |
| Musculoskeletal | 1.01 | 0.77-1.33 | 0.915 | 1.07 | 0.77-1.48 | 0.700 |
| Viral | 1.42 | 0.95-2.13 | 0.086 | 1.36 | 0.81-2.28 | 0.238 |

Adjusted for age, sex, comorbidities (microvascular disease [nephropathy, neuropathy, retinopathy], macrovascular disease [coronary artery disease, peripheral and cerebral vascular disease], heart failure, respiratory disease, dyslipidemia, fatty liver disease, and obesity), medications (acid inhibitors, respiratory system medications, anti-lipids and vaccines) and number of infections in the year prior to enrollment

**Appendix E:** **Odds Ratios (OR) Between Diabetes and Any Infection (Adjusted for Selected Covariates)**

| **Key Covariate** | **OR** | **95%CI** | **P Value** |
| --- | --- | --- | --- |
| **Crude** | 0.96 | 0.86-1.06 | 0.399 |
| **Age** | 1.02 | 0.91-1.14 | 0.726 |
| **Sex** | 0.99 | 0.89-1.1 | 0.838 |
| **Microvascular Disease** | 0.95 | 0.86-1.05 | 0.341 |
| **Macrovascular Disease** | 0.96 | 0.87-1.07 | 0.492 |
| **Age & Sex** | 1.05 | 0.94-1.17 | 0.378 |
| **Prior Infections** | 1.17 | 1.06-1.31 | 0.003 |
| **Backward Stepwise Selection Procedure:**  **Age, sex, smoking status, acid suppressing medications, respiratory medications, respiratory disease, prior infections** | 1.24 | 1.11-1.39 | <0.001 |
